# Transhepatic endovascular repair for portal vein haemorrhage

**DOI:** 10.1186/s42155-020-00149-8

**Published:** 2020-10-08

**Authors:** Lorenzo Carlo Pescatori, Hicham Kobeiter, Youssef Zaarour, Edouard Herin, Manuel Vitellius, Vania Tacher

**Affiliations:** 1 Assistance Publique - Hôpitaux de Paris (AP-HP), Service d’Imagerie Médicale, Hôpital Henri-Mondor, Créteil, France; 2 Université Paris-Est Créteil (UPEC), Créteil, France

**Keywords:** Portal stent, Trans-hepatic access, Closure device, Portal pseudoaneurysm

## Abstract

**Background:**

Post-surgical bleeding of the main portal vein (PV) is a rare event but difficult to manage surgically. Among the different options of treatment, endovascular stenting of the PV can be considered.

We reported two cases of stent-graft placement in PV with subsequent closure of the portal vein access with two percutaneous closure devices deployed simultaneously.

**Cases presentation:**

The first patient was a 43 years-old woman affected with a pseudoaneurysm of the extrahepatic PV, occurred after a duodenocephalopancreasectomy performed for a neuroendocrine tumour of the pancreatic isthmus. The second patient was a 54 years-old man suffering from multiple episodes of bleeding after liver transplantation, due to a PV fissure.

In both cases, a stent graft was placed into the portal system, between the PV and the superior mesenteric vein through a right trans-hepatic access to the portal system.

In both cases, a final control showed patency of the mesenteric vein and PV and no endoleak detection.

At the end of the procedure, two percutaneous closure devices were loaded, to close the transhepatic portal access. In one case, one of the devices did not work and the entry point was managed with a single device, without further complications.

No bleeding was seen though the entry point nor at the US examination performed right after the procedure.

After procedure, patients were prescribed with low-molecular weight heparin (LMWH) and kept under surveillance. For both patients, CT scan performed within 24h after the procedure, showed a patent stent-graft and no evidence of any venous portal ischemia.

The first patient was then transferred to another hospital, to continue observation and medical management. The second one underwent 2 months of hospitalization, during which he developed a pancreatic fistula and mild renal insufficiency. Then, he left the hospital to its native Country to continue his medical.

**Conclusion:**

PV stent-graft placement seems a feasible option to manage portal bleeding.

Trans-hepatic access is an easy and fast approach. The trans-hepatic portal accesses may be successfully managed with the deployment of percutaneous closure devices.

## Introduction

Bleeding of the main portal vein (PV) is a rare consequence of hepatobiliopancreatic surgery (Ginsburg et al. 2014) or traumatic events (Ierardi et al. 2016). As surgery on the PV is technically challenging (Fraga et al. 2009; Hyun et al. 2017), endovascular intervention could be a considered as an alternative (Ginsburg et al. 2014) and stent-graft placement should be preferred to simple embolization, in order to keep patency of the portal system and to avoid portal hypertension (Hyun et al. 2017).

Herein, we report two cases of stent-graft placement in PV with subsequent closure of the PV access with two percutaneous closure devices (Angioseal, Terumo, Tokyo, Japan) simultaneously deployed.

## Case report

### Patients

#### Considering the retrospective design of the paper, an ethical committee approval is not needed at our Institution

**Fig. 1:**
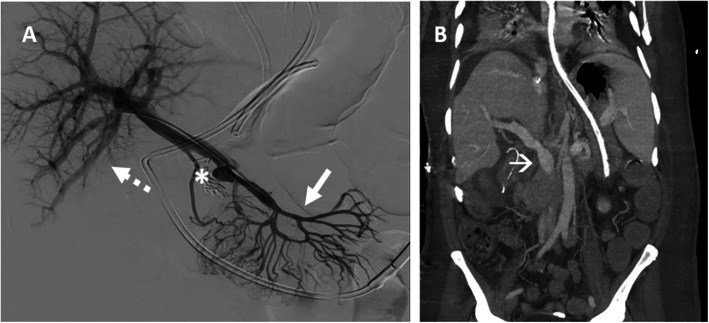
**a** Patient #1: Portography showing patent mesenteric vein (arrow) with the pseudoaneurysm at the porto-mesenteric confluence (asterisks), with a patent intra-hepatic portal vein system (dotted arrows). **b** Patient #2: CT scan showing irregularities of the extrahepatic wall of the PV (arrow), on the site of previous bleedings

Patient #1 was a 43 years-old woman referred to our Hospital because of a pseudoaneurysm of the extrahepatic PV, occurred after a duodenocephalopancreasectomy (DCP) performed elsewhere for a solitary neuroendocrine tumour of the pancreatic isthmus (Fig. 1a).

Patient #2 was a 54 years-old man with pancreatitis and multiple episodes of bleeding after a liver transplantation, because of primary sclerosing cholangitis. He was referred to endovascular treatment because a CT scan found irregularities of the extrahepatic wall of the PV, in contact with a peri-hepatic collection with traces of recent bleeding (Fig. 1b).

### Technique

In both cases, the intervention was planned under general anaesthesia. The patient was placed in a slight left-sided position. The 6th-segment branch of the PV was punctured with a 20-gauge Chiba needle (Cook, Bloomington, IN, USA), under ultrasound (US) guidance.

A 10 cm − 5 Fr sheath (Terumo, Tokyo, Japan) was introduced and a diagnostic portography was performed through a 4 Fr Vertebral catheter (Cordis, Milpitas, CA).

Then, the trans-hepatic access was progressively dilated and a stent graft was placed between the PV and the superior mesenteric vein.

**Fig. 2:**
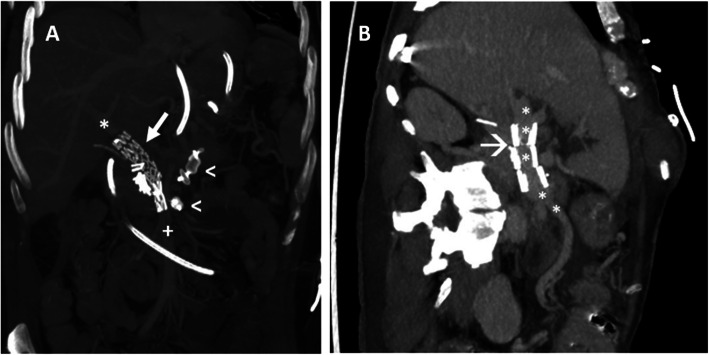
**a** Patient #1: Post-procedural CT scan showing a patent superior mesenteric vein (white cross) and portal vein (asterisk), with the stent-graft deployment (white arrow) and two vascular plugs (arrow-heads) at the splenic and inferior mesenteric vein origins. **b** Patient # 2: CT scan showing the correct deployment of the stent (arrow), with a patent portal vein (asterisks)

For patient #1,presenting with a PV of 12 mm of maximal diameter, a 16–13-56 mm stent-graft (Iliac stent graft, Zenith spiral Z, Cook) has been chosen and inserted through a 14 F sheat (Fig. 2a).

For patient #2, presenting with a PV of 16 mm of maximal diameter, a 20–59 mm stent-graft (iliac stentgraft, Zenith Alpha, spiral Z, Cook) has been used (Fig. 2b), inserted through a 16 Fr sheat.

For patient #1, before stent placement, two plugs of 16 mm and 10 mm in diameter [Amplatzer II, AVP II; St Jude Medical, St Paul, Minn], were deployed in the proximal splenic vein and in the proximal inferior mesenteric vein, respectively, in order to reduce the risk of post-procedural endoleaks (Fig. 2a). Conversely, no plugs have been used to plug the splenic vein in patient # 2, as it was not meant to be covered by the stent.

A final control performed though the 14 and 16 Fr sheats espectively, showed patency of the mesenteric and portal veins, with no endoleak, in both cases.

At the end of the procedure, two 0.035″ rigid guidewires (Advantage, Terumo) were placed in the superior mesenteric vein and two percutaneous devices (8 Fr-Angioseal, Terumo, Tokyo, Japan) were loaded, in order to close the trans-hepatic portal access.

**Fig. 3:**
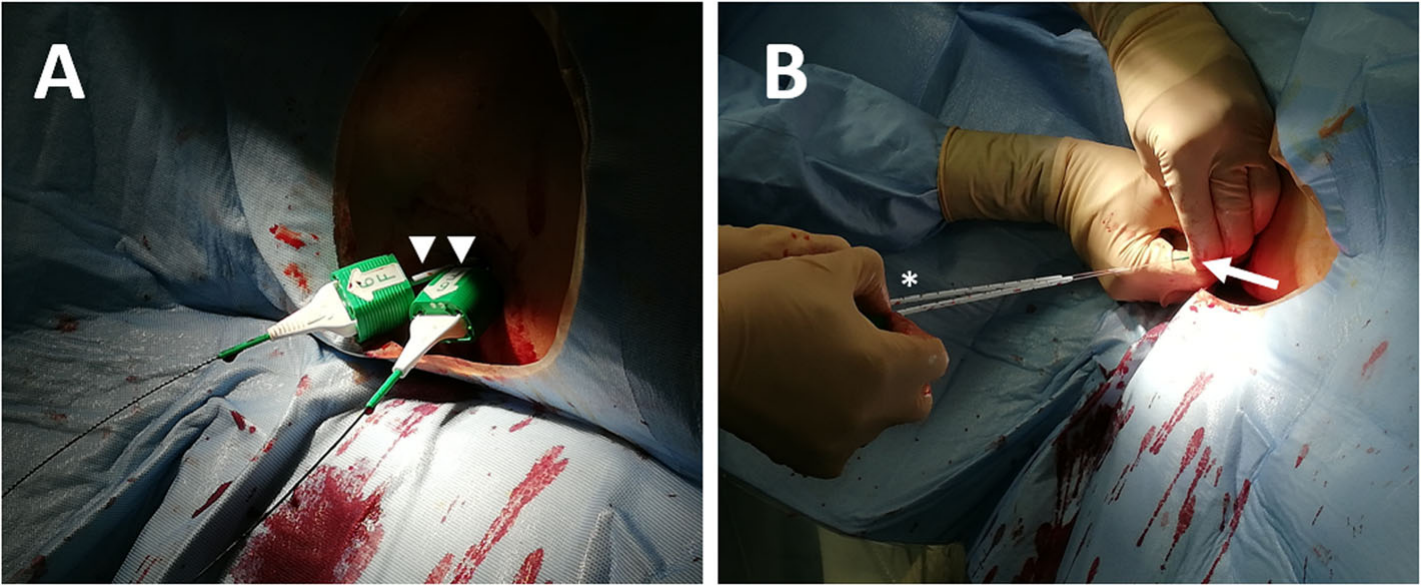
**a** Two parallel angioseals are inserted in the portal vein through the trans-hepatic access (arrowheads) on 0.035″ Advantage guidewires. **b** The Angioseals are deployed at the same time, with an operator keeping the tension of the suture stitches (asterisk) and the other one pushing the collagen plug within the liver parenchyma (arrow)

The two closure devices were deployed, while an operator was keeping the tension of the suture stitch and the other one was pushing the collagen plug within the liver parenchyma (Fig. 3).

In patient #2, one of the two percutaneous closure devices failed to grasp the PV.

Nevertheless, no bleeding was seen though the access and a post-procedural US confirmed the absence of extravasation.

Both patients were prescribed with low-molecular weight heparin (LMWH) and kept under surveillance.

For patient #1, a CT scan performed 12 h after the intervention, showed a patent stent-graft, with partial thrombosis of the proximal portions of the superior and inferior mesenteric veins, with an efficient collateral venous drainage with no venous ischemia of both spleen and bowel. The patient was then transferred to her original hospital, in order to continue observation and medical management.

For patient #2, a CT scan was performed 24 h after the procedure, showing a mild haematoma within the abdominal wall, without signs of active bleeding. In the subsequent 2 months of hospitalization, the patient developed a pancreatic fistula and mild renal insufficiency. Then, he left the hospital to its native Country to continue medical management.

## Discussion

We reported two cases of PV stent-graft placement through trans-hepatic access, that was closed with the deployment of two percutaneous closure devices at the end of the procedure.

To our best knowledge, no other cases of off-label use of arterial closure devices have been described in the international literature for this specific purpose, so far.

In both cases, PV damage was related to a previous major surgery of the upper abdomen. Nevertheless, despite arterial bleedings are relatively common after pancreatic or liver surgeries, PV haemorrhages are rare (Ginsburg et al. 2014; Ohnami et al. n.d.) and literature regarding endovascular management of extra-hepatic PV bleeding is mainly based on case reports.

Ginsburg et al. (2014) and Suzuki et al. (2015) reported cases of pancreaticoduodenectomy, complicated by a portal haemorrhage, successfully treated by PV stenting [iCAST stent (Atrium Medical Corporation, Hudson, USA) and Gore Excluder (W. L. Gore & Associates, Flagstaff, AZ, USA), respectively]. Walton et al. (2018) reported a PV stenting [FLUENCY plus (Bard Peripheral Vascular, Tempe, AZ, United States)] to manage a portal pseudoaneurysm caused by a bulging biliary stent, on a patient with B-cell lymphoma. All patients well recovered without any complication on the short-term (12 and 7 months, respectively).

Concerning post-traumatic bleedings, Ierardi et al. (2016) and Weber et al. (2016) reported two cases of patients presenting a PV haemorrhage after a car accident, successfully treated with a percutaneous covered stent [Ierardi et al.: Gore Excluder; Weber et al.: not reported].

But, as stenting of the portal vein needs a large transhepatic access, simple manual compression is not sufficient to manage the puncture site at the end of the procedure.

The trans-jugular approach would have been a valuable option to perform the intervention. Nevertheless, considering the risk of failure and the several punctures of the liver parenchyma to reach the PV as well as the risk of insufficient stability of the supporting system while deploying the stent, the trans-hepatic approach was preferred for performing the procedure.

Moreover, several techniques have been described to help the haemostasis though a trans-hepatic acces (i.e. collagen, coils, glue, gelfoam and plugs) (Dollinger et al. 2013; Adani et al. 2007; Wang et al. 2006).

But, when considering the large diameter of the accesses (i.e. 14 and 16 Fr) and the high volume of embolizing material that would have been needed to close them (with possible risks for foreign body infections or granulomas), the percutaneous closure devices have been preferred (Dollinger et al. 2013).

The percutaneous closure devices deployed were femoral closure devices, used since 1993 (de Swart et al. 1993). They are based on the compression of the site of puncture between a intravascular anchor and an absorbable extravascular plug (Aksoy et al. 2006), assuring a durable exclusion of the trans-arterial tract. They have been chosen for the small amount of embolizing material is left in place within the vessel and because the shaft of the system (12 cm) is long enough to ensure a satisfactory control during deployment, even for deep punctures (Menon et al. 2018).

But, as the sheaths used to perform the procedures were too large to be managed with a single percutaneous closure device, two devices have been deployed, in both patients, as already reported for arterial cases (Abi Rafeh et al. 2013).

In patient #2, one of the two percutaneous closure devices positioning failed. Nevertheless, a single device was enough to reduce the diameter of the portal access and to prevent the risk of a massive bleeding.

## Conclusion

PV stent-graft placement seems a feasible option to manage portal bleedings and pseudoaneurysms, in patients in whom surgical management is considered as life frightening event.

Trans-hepatic access is easy and fast approach to perform the procedure and our limited experience showed that even large portal accesses may be successfully managed with the deployment of percutaneous closure devices.

## Data Availability

Not applicable.
